# Crosstalk between the Unfolded Protein Response and NF-*κ*B-Mediated Inflammation in the Progression of Chronic Kidney Disease

**DOI:** 10.1155/2015/428508

**Published:** 2015-04-21

**Authors:** Zahraa Mohammed-Ali, Gaile L. Cruz, Jeffrey G. Dickhout

**Affiliations:** Department of Medicine, Division of Nephrology, McMaster University and St. Joseph's Healthcare Hamilton, 50 Charlton Avenue East, Hamilton, ON, Canada L8N 4A6

## Abstract

The chronic inflammatory response is emerging as an important therapeutic target in progressive chronic kidney disease. A key transcription factor in the induction of chronic inflammation is NF-*κ*B. Recent studies have demonstrated that sustained activation of the unfolded protein response (UPR) can initiate this NF-*κ*B signaling phenomenon and thereby induce chronic kidney disease progression. A key factor influencing chronic kidney disease progression is proteinuria and this condition has now been demonstrated to induce sustained UPR activation. This review details the crosstalk between the UPR and NF-*κ*B pathways as pertinent to chronic kidney disease. We present potential tools to study this phenomenon as well as potential therapeutics that are emerging to regulate the UPR. These therapeutics may prevent inflammation specifically induced in the kidney due to proteinuria-induced sustained UPR activation.

## 1. Introduction

Chronic kidney disease (CKD) is a major healthcare concern in the population and its treatment places a large economic burden on healthcare systems. The prevalence of CKD is growing rapidly in North America, thereby increasing cost of hemodialysis and the number of patients requiring organ transplants [1, 2]. CKD can be defined as kidney damage lasting over 3 months caused by structural or functional abnormalities of the kidney, with or without decreased glomerular filtration rate (GFR), that can lead to decreased GFR [3]. Immune cell activation and inflammatory responses are important factors in the development of both acute and chronic kidney diseases [4]. Therefore, immunomodulation therapy may be an important new avenue to help prevent the progression of CKD, reducing patient morbidity and mortality and its associated economic costs.

Endoplasmic reticulum (ER) stress is a cellular pathology that occurs due to an imbalance between protein folding capacity and protein folding demand [5, 6]. The accumulation of misfolded proteins in the ER results from disturbances in ER homeostasis making conditions unfavourable for protein folding, or, mutations in proteins that impair their proper folding [6]. These disturbances in ER homeostasis include hypoxia, glucose depletion, and oxidative stress [7]. ER stress plays an important role in the pathogenesis of chronic diseases associated with the accumulation of misfolded proteins. These include neurodegenerative diseases such as Alzheimer's and Parkinson's diseases, atherosclerosis, diabetes mellitus and chronic kidney disease [8–10]. ER stress results in the activation of the unfolded protein response (UPR), an evolutionarily conserved cellular response regulated primarily by glucose regulated protein 78-KD (GRP78). The UPR involves the activation of processes such as apoptosis and inflammation that determine the fate of cell survival and tissue scarring [6, 8, 11]. Therefore, ER stress is an important factor in the development of renal diseases and the study of the UPR pathways is likely to reveal molecular targets that influence disease progression.

In this review, we will highlight the current literature on inflammatory and ER stress responses in CKD and then elucidate pathways of ER stress-mediated inflammation that link these two processes. ER stress-mediated inflammation has been implicated in the pathogenesis and development of several diseases including obesity, type 2 diabetes, inflammatory bowel disease, atherosclerosis, cystic fibrosis, and cancer [11]. Nuclear factor-*κ*B (NF-*κ*B) plays an important role in renal inflammation in human and experimental models of kidney disease [12, 13] and is the main transcription factor in ER stress-mediated inflammation [11, 14, 15]. Therefore, we suggest that ER stress-mediated inflammation could be a key process in CKD development. The study of pathways involved in ER stress-induced inflammation may give rise to highly specific molecular targets that can impact the outcome of CKD progression.

## 2. Inflammation in Kidney Disease

CKD can lead to end stage renal failure (ESRF), an important comorbidity of cardiovascular disease [16]. Human CKD studies have indicated a relationship between hypertension and renal diseases [17, 18]. Renal T cell and macrophage infiltration has been established in experimental models of hypertension such as in spontaneously hypertensive rats [19, 20], Dahl salt-sensitive rats [21–23], Angiotensin (Ang) II-induced hypertension [24–26], and DOCA-salt hypertension [26–28]. The administration of the immunosuppressive drug, mycophenolate mofetil, appeared to reduce renal inflammation, mesangial cell and macrophage infiltration, oxidative stress, and tubulointerstitial damage in animal models of hypertension [29–31]. Previous reports have shown an improvement in renal function and a reduction in tubular necrosis and immune cell infiltration with T cell deficiency in mouse models of renal ischemic injury [32, 33]. Moreover, in a study by Moon et al., intrarenal CD3^+^ T cells were increased in proteinuric mice with streptozotocin-induced diabetes [34]. Type 2 diabetic patients also experienced an infiltration of CD4^+^, CD8^+^, and CD20^+^ cells in the renal interstitium where higher levels of CD4^+^ and CD20^+^ cells correlated with proteinuria [34]. Therefore, T cells appear to play a role in the development of renal damage in various models of kidney disease. Likewise, macrophage infiltration has been well-established as a prominent characteristic of tubulointerstitial damage that occurs in kidney disease irrespective of the origin [35, 36]. Monocyte chemoattractant protein-1 (MCP-1) is a chemokine known for recruiting macrophages and lymphocytes to sites of tissue injury and infection [37]. Blockade of MCP-1 or its receptor CCR2 has been shown to attenuate macrophage infiltration, renal fibrosis, and tubulointerstitial injury [38–40]. MCP-1 expression has been demonstrated in membranous nephropathy, as an indicator of progression to ESRF [41] and in human crescentic glomerulonephritis where it has been associated with glomerular macrophage infiltration [42]. Also, Urinary excretion of MCP-1 is observed in a majority of adult patients with autosomal dominant polycystic kidney disease [43]. Collectively, these studies suggest an important role for T cells and macrophages in the development and progression of CKD.

Reports have pointed to the significant contribution of the transcription factor, NF-*κ*B, in the pathogenesis of proteinuric renal damage [44–46]. NF-*κ*B is present in an inactive form in all cell types and is released from an inhibitory subunit (I*κ*B) upon stimulation. When released, NF-*κ*B activates the transcription of a large number of target genes: RANTES (regulated upon activation normal Tcell expressed and secreted), interleukin (IL)-1, IL-2, IL-6, MCP-1, tumour necrosis factor- (TNF-) *α*, adhesion molecules, and several other proinflammatory mediators [47]. The inhibition of TNF-*α*, an NF-*κ*B-inducible gene, has been shown to delay the progression of hypertension and renal damage by reducing proteinuria, urinary MCP-1 excretion, and renal macrophage infiltration in animal models of DOCA salt and Ang II-dependent hypertension [48, 49]. Renal NF-*κ*B expression and/or activation was observed in patients with glomerulonephritis [50, 51], diabetic nephropathy [52], and proteinuric renal disease (minimal change disease and membranous nephropathy) where it correlated with the magnitude of proteinuria [46].

Studies on CKD have established proteinuria as an important modifiable factor in disease progression [53]. Kidney disease patients with a higher mean baseline proteinuria ≥3.0 g/day demonstrated a greater drop in GFR over time [54]. Therefore, the higher the urinary protein excretion values, the faster the decline in GFR and disease progression, making it an important predictor of ESRF independent of the nature of the underlying disease [55]. Protein overload models* in vitro* using albumin and IgG resulted in an increase in NF-*κ*B activity accompanied by upregulation in NF-*κ*B-inducible genes RANTES and MCP-1 [56]. Cultured rat tubular epithelial cells treated with bovine serum albumin (BSA) showed significant NF-*κ*B activation in a time- and dose-dependent manner [57]. In a rat model of nephritis using uninephrectomy and BSA-overload, nephritic rats experienced an increase in NF-*κ*B activity that correlated with augmentation in urinary protein excretion [57]. This correlation is also observed in rat models of immune complex glomerulonephritis [58], passive Heymann nephritis [59], and renal mass reduction [59]. This finding suggests that NF-*κ*B activation may lead to proinflammatory gene expression and recruitment of inflammatory cells in response to proteinuria. Modulation of NF-*κ*B activity has been performed experimentally [60, 61]. The administration of a recombinant adenovirus vector expressing a truncated form of I*κ*B*α* in a rat model of tubulointerstitial injury (TI) was able to specifically inhibit NF-*κ*B activation [60]. NF-*κ*B inhibition in this model was able to attenuate proteinuria-induced TI injury as well as TGF-*β*, VCAM-1, and fibronectin expression in TI lesions [60]. Parthenolide and gliotoxin are able to block NF-*κ*B DNA-binding by stabilizing I*κ*B*α*. The use of these drugs in experimental models of mesangial proliferative glomerulonephritis leads to lower activation of NF-*κ*B in the glomeruli and tubules of nephritic animals [61]. These agents resulted in decreased proteinuria and glomerular lesions as well as downregulated inflammatory responses and reduced glomerular recruitment of mononuclear cells [61]. Pathways regulated by NF-*κ*B are, therefore, essential in the development of inflammation and proteinuric renal damage in various human and experimental models of kidney disease.

## 3. ER Stress in Kidney Disease

ER stress leads to the activation of the UPR. The UPR includes the dissociation of GRP78 from three ER transmembrane proteins: PKR (double-stranded RNA-dependent protein kinase) like ER protein kinase (PERK), inositol-requiring enzyme 1 (IRE1), and activating transcription factor 6 (ATF6) [6, 62, 63]. UPR activation causes the release of ATF6 leading to its cleavage in the Golgi by site 1 and site 2 proteases [64, 65]. The cytosolic, DNA-binding fragment of ATF6 travels to the nucleus where it activates the transcription of ER chaperones, enzymes that aid in protein folding and secretion as well as components of ER-associated degradation [62]. PERK is a Ser/Thr protein kinase and is activated through homodimerization and transphosphorylation during the UPR. PERK activation leads to the phosphorylation and inhibition of eIF2*α*, a component of the translation initiation complex [66, 67]. This pathway reduces the recognition of initiation AUG codon thereby attenuating translation to decrease protein load on the ER. Certain mRNAs with short open reading frames in the 5′-untranslated region are preferentially translated in this pathway including ATF4 [66, 67]. ATF4 is a transcription factor that induces the expression of ER stress target genes, notably CHOP, resulting in apoptosis [6, 62]. CHOP is a transcription factor that upregulates expression of proapoptotic factors and decreases antiapoptotic genes such as Bcl2 [6, 62]. IRE1*α* activation results in X-box binding protein-1 (XBP1) mRNA splicing. This step causes a change in the reading frame allowing the translation of a transcription factor that induces expression of genes with an ER stress response element including ER chaperons such as GRP78 [62, 68–70]. The IRE1*α* arm of the UPR may also stimulate proapoptotic pathways via its activation of Caspase 12 (rodents)/Caspase 4 (humans) and c-Jun N-terminalkinase (JNK) phosphorylation [62, 68–70].

Kidney biopsies from patients at different stages of glomerulonephritis showed upregulation of GRP78 and CHOP highlighting the significance of ER stress responses in renal disease progression [71]. The upregulation of ER stress markers has also been shown in nephrotic syndrome patients [72]. ER stress pathways may be targeted pharmacologically to attempt to modify the outcome of renal disease [62]. The use of 4-phenylbutyric acid (4-PBA), a low molecular weight chemical chaperone, prevented ER stress in a rat model of streptozotocin-induced diabetic nephropathy and also significantly reduced urinary protein excretion [73]. Treatment with 4-PBA in this model also reduced basement membrane thickening, mesangial cell proliferation, and mesangial matrix accumulation in rats with diabetic nephropathy [73]. Similarly, 4-PBA was able to cause a significant decrease in fibrosis as shown by marked reduction in collagen type I, fibronectin, and *α*-SMA in rats undergoing the unilateral ureteral obstruction model [74].

In a model of acute kidney injury, 4-PBA treatment prevented damage to the outer medullary stripe of the kidney and reduced ER stress upregulation and CHOP-induced apoptosis [75]. Further, the deletion of the GRP78 ER retention sequence, KDEL, has been shown to exacerbate renal injury by increased urinary protein excretion and tubular damage in an* in vivo* model of albumin overload [76]. Therefore, the manipulation of the UPR has a significant impact on CKD progression.

## 4. Interrelationship between Inflammatory Pathways and ER Stress in Kidney Disease

All three arms of the UPR, IRE1*α*, PERK, and ATF6 result in transcriptional activation of proinflammatory genes by primarily activating NF-*κ*B [14, 15, 77]. In mammals, the NF-*κ*B family consists of five members: p65/RelA, cRel, RelB, p100/p52, and p105/p50 [12]. These proteins can homodimerize and form heterodimers with each other and share a highly conserved domain, Rel homology region (RHR) [12, 78, 79]. NF-*κ*B dimers are bound to inhibitory I*κ*B proteins in the cytoplasm and are inactive since I*κ*B interferes with the function of the nuclear localization sequence present on the RHR domain [80]. Phosphorylation and subsequent degradation of I*κ*B is required for NF-*κ*B translocation to the nucleus [78].

**Figure 1:**
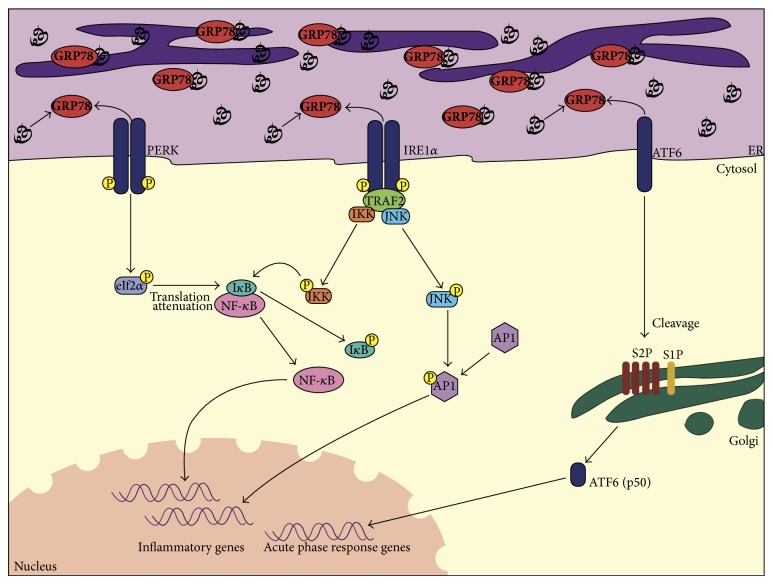
Overview of UPR-induced inflammatory gene transcription. The dissociation of GRP78 from the transmembrane transducers, PERK, IRE1*α*, or ATF6, leads to their activation. PERK activation brought about by autophosphorylation results in the phosphorylation of eIF2*α* and general translation attenuation reducing the I*κ*B available to bind to NF-*κ*B. Due to I*κ*B's shorter half-life, more NF-*κ*B is free to enter the nucleus and activate transcription of inflammatory genes. Autophosphorylation of IRE1*α* causes the cytosolic domain to associate with TRAF2. The IRE1*α*-TRAF2 complex recruits IKK which phosphorylates I*κ*B resulting in NF-*κ*B activation. This complex recruits protein kinase JNK leading to phosphorylation of transcription factor AP1 as well. Upon activation, ATF6 leaves the ER and undergoes cleavage by site 1 (S1P) and site 2 proteases (S2P) in the Golgi complex. The 50-kilodalton cleavage product (p50) acts as a transcription factor in the nucleus and results in the transcriptional initiation of acute phase inflammatory response genes.

Figure 1 shows an overview of the pathways involved in ER stress-mediated activation of inflammatory gene transcription. During prolonged ER stress, the dissociation of GRP78 results in autophosphorylation of IRE1*α* causing a conformational change in its cytosolic domain. The cytosolic domain of activated IRE1*α* then binds to adaptor protein TNF*α* receptor-associated factor 2 (TRAF2) [81]. The IRE1*α*-TRAF2 complex recruits I*κ*B kinase (IKK), phosphorylating I*κ*B resulting in its degradation and NF-*κ*B activation [82, 83]. IRE1*α*-TRAF2 complex can also recruit protein kinase JNK leading to phosphorylation of transcription factor AP1, linking ER stress to other proinflammatory pathways [84]. UPR activation of PERK results in translation attenuation via phosphorylation of eIF2*α*, a component of the translation initiation complex. This process causes decreased translation of I*κ*B, freeing more NF-*κ*B to translocate to the nucleus [85, 86]. ATF6 leaves the ER upon activation and undergoes cleavage by site 1 and site 2 proteases in the Golgi complex. These activated ATF6 fragments form homodimers and induce transcription of acute-phase response genes [14, 64, 65]. Although the ATF6 pathway can also result in NF-*κ*B activation via phosphorylation of AKT [87], the PERK and IRE1*α* arms of the UPR have been demonstrated as crucial for ER stress-induced NF-*κ*B activation. In a study by Kaneko et al. [82], human embryonic kidney (HEK) 293T cells treated with thapsigargin (TG) showed phosphorylation and degradation of I*κ*B and upregulation of NF-*κ*B. This effect was suppressed with transfection of a dominant-negative mutant of IRE1*α* or a dominant-negative mutant of TRAF2 [82]. ER stress-induced activation of NF-*κ*B using thapsigargin and tunicamycin was impaired with IRE1*α* knockdown and IRE1*α* deficiency in mouse embryonic fibroblasts (MEFs) [83]. Reconstitution of IRE1*α* deficient MEFs with IRE1*α* resulted in the recovery of ER stress-induced NF-*κ*B activation [83]. Similarly, studies have shown eIF2*α* phosphorylation results in NF-*κ*B activation through translation attenuation as it occurs independently of I*κ*B phosphorylation or degradation [85, 86]. Moreover, PERK deficiency and eIF2*α* mutant MEFs inhibited NF-*κ*B activation in response to treatment with ER stress inducing agents indicating that PERK-induced eIF2*α* phosphorylation is essential in NF-*κ*B activation [85].

## 5. Investigating ER Stress-Induced Inflammation in the Kidney

Since ER stress pathways and inflammatory responses have been demonstrated in human CKD as well as in animal models of the disease, the use of pharmacological tools* in vitro* and* in vivo* may help elucidate molecular targets essential to disease progression.  Table 1 summarizes drugs that could be used to manipulate the UPR.

Salubrinal (Sal) is a small molecule identified through high-throughput screening for its ability to enable cells to withstand ER stress. This drug acts as a phosphatase inhibitor specific in preventing the activation of eIF2*α*, a key component of the PERK pathway [88].* In vivo*, Sal has been shown to protect against cyclosporine A-induced nephrotoxicity [89], which has been associated with ER stress [90]. GSK2606414 is a potent and selective PERK inhibitor shown to inhibit PERK activation in response to ER stress in A549 cells, a human lung adenocarcinoma cell line, and inhibits the growth of human tumor xenografts in mice [91]. Since it penetrates the blood-brain barrier, GSK2606414 administration has been shown to impart neuroprotective effects and prevent clinical disease in prion-infected mice [92]. Therefore, Sal and GSK2606414 provide pharmacological interventions that could be used to study the effect of the PERK pathway on ER stress-induced inflammation.

STF 083010 is a novel molecule, first identified though high-throughput screening. This reagent was able to inhibit IRE1*α* endonuclease activity during ER stress both* in vitro* and* in vivo*. Pretreatment of RPMI 8226 human multiple myeloma cells with STF 083010 blocked XBP1 splicing activated by both tunicamycin- and thapsigargin-induced ER stress. Additionally, treatment of XBP1-luciferase reporter mice with STF 083010 reduced XBP1 splicing in an* in vivo* model of ER stress induced by bortezomib [93]. Irestatin is another molecule that is able to inhibit IRE1*α* endonuclease activity and has been reported to disrupt the growth of malignant myeloma cells [94]. Therefore, STF 083010 as well as Irestatin could be used as pharmacological tools to study the effect of inhibiting the IRE1*α* pathway on ER stress-induced inflammation.

4-(2-Aminoethyl) benzenesulfonyl fluoride (AEBSF) has been shown to prevent ER stress-induced cleavage of ATF6*α* and ATF6*β*, resulting in inhibition of transcriptional induction of ATF6-target genes [95]. This compound has been used as a serine protease inhibitor in both* in vitro* [96, 97] and* in vivo* studies [98, 99]. Therefore, AEBSF could be used as a pharmacological tool to study the effect of inhibiting the ATF6 pathway on ER stress-induced inflammation.

4-PBA is a chemical chaperone that has been shown to stabilize protein conformation and improve protein folding in the ER by inhibiting ER stress. This drug has been used clinically for the treatment of urea cycle disorders in children, sickle cell disease, thalassemia and cystic fibrosis [100]. In particular,* in vitro* studies have shown that 4-PBA administration results in a reduction in GRP78 levels in response to ER stress [101, 102]. In a mouse model of brain ischemia, pretreatment or posttreatment with 4-PBA at therapeutic doses was able to attenuate disease progression possibly as a result of a decrease in protein load retained by the ER [103]. Tauroursodeoxycholate (TUDCA), a derivative of an endogenous bile acid, is another chaperone that has been shown to resolve ER stress in liver and adipose tissue thereby normalizing hyperglycemia and restoring systemic insulin sensitivity in obese and diabetic mice [104]. TUDCA has also been shown to inhibit the expression of ER stress markers in intestinal epithelial cells [105] and attenuate intestinal inflammation in a rodent model of inflammatory bowel disease [106]. Therefore, both 4-PBA and TUDCA could be used to manipulate ER stress responses.

ER stress-inducing agents tunicamycin (TM), thapsigargin (TG), and indoxyl sulfate (IS) could be used to evaluate the effects of ER stress induction* in vitro* and* in vivo*. TM is a nucleoside antibiotic, which inhibits N-linked protein glycosylation, and is used to model acute kidney injury* in vivo* [75]. TG is a plant-derived sesquiterpene lactone and induces ER stress by inhibiting the sarcoplasmic/endoplasmic reticulum calcium ATPase (SERCA) pump and altering Ca^2+^ homeostasis [107]. Both TG and TM result in upregulation of ER stress markers GRP78, GRP94, CHOP, and phosphorylated eIF2*α* in human proximal tubule cells [75, 108, 109]. IS is a uremic toxin that has been reported to accumulate in the serum of CKD patients and contribute to disease progression [110, 111]. IS interacts with organic anion transporter types 1 and 3 and is therefore able to incorporate into the basolateral membrane of renal proximal tubule cells [112]. IS was shown to induce ER stress via oxidative stress in human proximal tubule cells and inhibits cell proliferation through the upregulation of CHOP and ATF4 in these cells [113].

**Figure 2:**
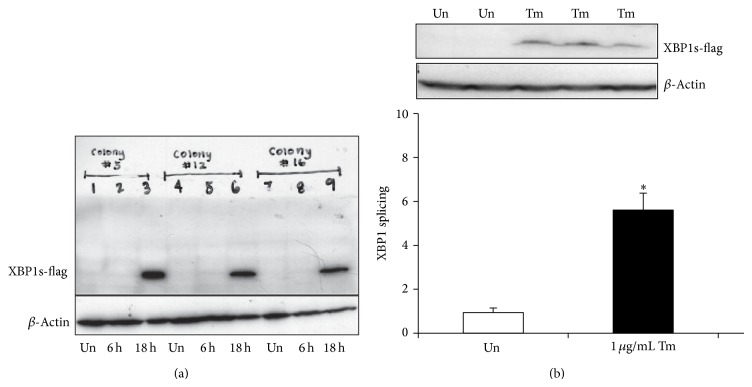
Detection of XBP1 splicing in human proximal tubule-2 cells using XBP1sVenus reporter. (a) XBP1sVenus was placed in a hygromycin B selectable cassette and transfected into HK-2 cells. Cells were then selected for stable incorporation of XBP1sVenus with 0.5 mg/mL hygromycin B. Clones were screened with 1 *μ*g/mL tunicamycin (Tm) for 6 or 18 hours. Clones 3, 12, and 16 were found to give robust responses when probed with the flag-tag antibody and produced green fluorescence. (b) Clone 12 from the stably transfected XBP1s HK-2 reported cell line was grown up and untreated (Un) or treated with 1 *μ*g/mL tunicamycin (Tm). The expression of the flag-tag shows IRE1 activation in the reporter cell line.

Human kidney proximal tubule cells (HK2 cell line) [114] could be used to generate a reporter cell line for XBP1 splicing. A previously published tool for monitoring XBP1 splicing involves an ER stress-activated indicator (ERAI) [115]. The ERAI consists of a F-XBP1ΔDBD-venus plasmid, a variant of green fluorescent protein, fused as a reporter downstream of a partial sequence of human XBP1 containing the 26-nucleotide IRE1 splicing site [115]. The plasmid also contains a hygromycin B resistance gene. We utilized hygromycin B (0.5 mg/mL) to select HK2 cells transfected with this plasmid to generate a stable cell line. The ERAI contains both a fluorescent reporter (venus) and a flag-tag. Thus, it can be used to detect XBP1 splicing by immunofluorescence and Western blotting.  Figure 2 demonstrates our selection of HK2 cells stably expressing ERAI and the detection of XBP1 splicing in these cells in response to TM using Western blotting for the flag-tag. This tool could be used to detect IRE1 activity to evaluate its effect on inflammatory processes in the kidney. The reporter construct has been employed in transgenic mice to allow the detection of IRE1 activity in whole animals [115]. This tool would be generally applicable to various models of CKD.

A number of pharmacological and genetic tools are available to study ER stress-induced inflammation in the kidney both* in vivo* and* in vitro*. Manipulation of ER stress pathways will provide insight about the influence of the UPR on the inflammatory response and might reveal useful and more specific targets in key processes during CKD development.

## 6. Conclusion

ER stress-mediated inflammation appears to be important in the progression of CKD. Further, specific pathways within the UPR response result in ER stress-induced inflammation. Thus, the targeted pharmacological manipulation of the UPR holds promise to selectively inhibit the inflammatory consequences of UPR activation without disrupting proteostasis.

## Figures and Tables

**Table 1:** Pharmacological manipulation of the UPR. To study the various pathways of the UPR, pharmacological manipulations to the specific pathways can be utilized. To examine the PERK pathway, salubrinal is an inhibitor of the dephosphorylation of eIF2*α*. To investigate the IRE1 pathway, STF-083010 and Irestatin are inhibitors of IRE1 endonuclease activity. The ATF6 pathway can be inhibited with 4-(2-aminoetheryl) benzenesulfonyl fluoride (AEBSF) to prevent cleavage of ATF6. The role of protein folding chaperones can be determined by utilizing artificial chaperones including 4-phenylbutyrate (4-PBA) and tauroursodeoxycholic acid (TUDCA), which aid in the folding of proteins. Further, to investigate sustained activation of the unfolded protein response, classic ER stress inducers, tunicamycin and thapsigargin, can be used as well as disease-related inducers including indoxyl sulfate.

UPR gene	Pharmacological manipulation	Description
PERK	Salubrinal	Phosphatase inhibitor prevents dephosphorylation of eIF2*α*
	GSK2606414	Potent and selective PERK inhibitor

IRE1*α*	STF083010	Specifically inhibits IRE1*α* endonuclease activity during ER stress without affecting its kinase activity
	Irestatin	Specific inhibitor of IRE1*α*

ATF6	4-(2-Aminoethyl) benzenesulfonyl fluoride (AEBSF)	Serine protease inhibitor inhibits site 1 and site 2 proteases preventing ATF6 cleavage and inhibits transcription of ATF6 target genes

Small chemical protein folding chaperones	4-Phenylbutyrate (4-PBA)	4-PBA and TUDCA aid in protein folding reducing misfolded protein accumulation in the ER
	Tauroursodeoxycholic acid (TUDCA)	

UPR activating agents	Tunicamycin	Inhibitor of N-linked protein glycosylation hinders a process required for proper protein folding
	Thapsigargin	Inhibitor of sarcoplasmic/endoplasmic reticulum calcium ATPase (SERCA) pump causes ER stress
	Indoxyl sulfate	Uremic toxin that causes ER stress via oxidative stress
